# Co-Occurring Gland Angularity in Localized Subgraphs: Predicting Biochemical Recurrence in Intermediate-Risk Prostate Cancer Patients

**DOI:** 10.1371/journal.pone.0097954

**Published:** 2014-05-29

**Authors:** George Lee, Rachel Sparks, Sahirzeeshan Ali, Natalie N. C. Shih, Michael D. Feldman, Elaine Spangler, Timothy Rebbeck, John E. Tomaszewski, Anant Madabhushi

**Affiliations:** 1 Case Western Reserve University, Department of Biomedical Engineering, Cleveland, Ohio, United States of America; 2 Rutgers, the State University of New Jersey, Department of Biomedical Engineering, Piscataway, New Jersey, United States of America; 3 University of Pennsylvania, Department of Pathology and Laboratory Medicine, Philadelphia, Pennslyvania, United States of America; 4 University of Pennsylvania, Department of Clinical Epidemiology and Biostatistics, Philadelphia, Pennslyvania, United States of America; 5 University at Buffalo, State University of New York, Department of Pathology and Anatomical Sciences, Buffalo, New York, United States of America; UT MD Anderson Cancer Center, United States of America

## Abstract

Quantitative histomorphometry (QH) refers to the application of advanced computational image analysis to reproducibly describe disease appearance on digitized histopathology images. QH thus could serve as an important complementary tool for pathologists in interrogating and interpreting cancer morphology and malignancy. In the US, annually, over 60,000 prostate cancer patients undergo radical prostatectomy treatment. Around 10,000 of these men experience biochemical recurrence within 5 years of surgery, a marker for local or distant disease recurrence. The ability to predict the risk of biochemical recurrence soon after surgery could allow for adjuvant therapies to be prescribed as necessary to improve long term treatment outcomes. The underlying hypothesis with our approach, co-occurring gland angularity (CGA), is that in benign or less aggressive prostate cancer, gland orientations within local neighborhoods are similar to each other but are more chaotically arranged in aggressive disease. By modeling the extent of the disorder, we can differentiate surgically removed prostate tissue sections from (a) benign and malignant regions and (b) more and less aggressive prostate cancer. For a cohort of 40 intermediate-risk (mostly Gleason sum 7) surgically cured prostate cancer patients where half suffered biochemical recurrence, the CGA features were able to predict biochemical recurrence with 73% accuracy. Additionally, for 80 regions of interest chosen from the 40 studies, corresponding to both normal and cancerous cases, the CGA features yielded a 99% accuracy. CGAs were shown to be statistically signicantly (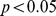) better at predicting BCR compared to state-of-the-art QH methods and postoperative prostate cancer nomograms.

## Introduction

Each year in the United States, nearly 60,000 men diagnosed with prostate cancer (CaP) undergo radical prostatectomy (RP) [1]. In cases for which there is no prior evidence of disease spread, treatment of CaP with RP has generally resulted in favorable long term outcome [2]. However, for 15–40% of RP patients, biochemical recurrence (BCR) occurs within 5 years of surgery [1]. BCR is commonly defined as a detectable persistence of prostate specific antigen (PSA) of at least 0.2 ng/ml and is suggestive of either local or distant recurrence of disease necessitating further treatment [3]. Consequently, it is important to be able to predict the risk of BCR soon after surgery, so that if needed, adjuvant treatments can be initiated.

Gleason scoring [4] is a pathology based grading system based on the visual analysis of glandular and nuclear morphology. Low Gleason scores have been associated with more favorable longer term prognosis for prostate cancer, while the converse is true for higher Gleason scores [5]. Gleason scoring combines the grade of the most common and second most common patterns within the tissue section, resulting in a Gleason sum ranging from 2 (least aggressive) to 10 (most aggressive). Gleason score is currently regarded as the best biomarker for predicting disease aggressiveness and longer term, post-surgical patient outcome [4]. Unfortunately, post-surgical outcome of prostate cancer patients with intermediate Gleason scores can vary considerably [6]. Some statistical tables suggest a 5-year BCR-free survival rate as low as 43% in men with Gleason sum 7 [5]. Furthermore, Gleason scoring is subject to considerable inter-reviewer variability [7]. Allsbrook et al. [7] reported a kappa-coefficient of 0.4 representing moderate agreement amongst pathologists for grading Gleason score 7 patterns. Therefore, the prognostic value of Gleason scoring alone for predicting BCR in RP patients with intermediate Gleason scores appears to be limited.

Over the last two decades, many postoperative nomograms have been developed to incorporate additional clinical variables such as tumor stage, pre-operative PSA, or positive surgical margins [5], [8]–[11] in order to predict patient and disease outcome. The Kattan nomogram [8] incorporated these parameters to predict 80 month BCR free survival following radical prostatectomy. Han et al. [5] incorporated Gleason sum, tumor stage, and pre-operative PSA into a series of probability tables, known as the Han Tables. Subsequently, the Stephenson nomogram [9] added the date of surgery as a prognostic variable. The University of California at San Francisco built their own risk score predictor (CAPRA) [10] to separate postoperative CaP patients into low, medium, and high risk categories. Apart from the parameters used in the Kattan nomogram, the CAPRA score also included the percentage of positive biopsy cores into their risk assessment. Hinev et al. [11] performed an independent study advocating the use of the Memorial Sloan Kettering Cancer Center (MS-KCC) nomogram, developed by Kattan and Stephenson, suggesting superior prediction of 5-year BCR compared to the Han tables. The MS-KCC nomogram adds additional variables such as age and time free of cancer. These nomograms represent the state-of-the-art in postoperative CaP prediction of BCR, but still rely heavily on Gleason score, which is derived from pathologist interpretation.

The recent advent of digital whole slide scanners has allowed for high resolution digitization of tissue slides. These digitized slide images can be subsequently subjected to quantitative histomorphometry (QH). A variety of QH tools have been previously employed for describing, classifying, and diagnosing disease patterns from histopathology images [12]. In the context of excised prostate pathology specimens, QH has been utilized successfully in a wide range of applications from cancer detection to prognosis, Monaco et al. developed and employed a markov random field (MRF) algorithm for detection of prostate cancer [13].

Some researchers have also explored the role of image texture in characterizing the appearance of CaP morphology. For the purpose of automated CaP grading, Jafari-Khouzani et al. [14] examined the role of second order image intensity texture features based on co-occurrence matrices. Co-occurrence matrices evaluate the frequency with which two image intensities appear within a pre-defined distance of each other within a neighborhood. A series of second-order statistical features (e.g. entropy) [15] to describe the co-occurrence matrix can then be extracted and serve to describe the local image texture. Other texture features such as first order statistical image intensity and steerable gradient filters (e.g. Gabor filters) [16] have also been used to predict CaP. Texture features, while they have been shown to be useful for characterizing CaP morphology, often suffer from a lack of transparency and interpretability.

Another class of approaches have attempted to explicitly model CaP appearance by interrogating the spatial arrangement of individual nuclei and glands. Tabesh et al. [17] investigated color, texture, and structural morphology to perform automated Gleason scoring in prostate histopathology. In Doyle et al. [16], morphological descriptors such as gland size and perimeter ratio were shown to distinguish benign and malignant histological regions. Veltri et al. [18] investigated nuclear morphology using a descriptor called nuclear roundness variance to predict biochemical recurrence in men with prostate cancer.

Many researchers have also attempted to model QH tissue architecture, via the use of graphs networks to characterize the spatial arrangement of nuclei and glands [16], [19]–[21]. Christens-Barry et al. used Voronoi- and Delaunay-based graph tessellations to describe tissue architecture in CaP histology [19]. Doyle et al. [16] showed that the Minimum Spanning Trees, in addition to Voronoi, Delaunay features appeared to be strongly correlated to Gleason grade. However, these features are derived from fully connected graphs. This approach suggests that nuclei embedded within stromal and epithelial regions will be connected via these graphs and hence the graph edges will traverse the epithelial stromal interfaces and regions [22]. Hence the features extracted from Voronoi or Delaunay graphs represent the “averaged” attributes of both stromal and epithelial architecture, and thus overlooks the local contributions of stroma and epithelium independently within the graphs. Alternatively, analysis of local subgraphs [20], [21], [23], [24], which unlike global graphs (e.g. Voronoi and Delaunay) that aim to capture a global architectural signature for the tumor, can allow for quantification of local interactions within flexible localized neighborhoods. Gunduz et al. [23] noted a natural clustering of cells and utilized cell graphs to model gliomas and differentiate cancerous, healthy, and non-neoplastic inflamed tissue. Demir et al. [25] and others [20], [23], [24] have developed a set of graph features to quantify the local cell-graphs. Bilgin et al. [20] similarly extracted features from different types of local cell graphs for classification of breast tissue. Features were extracted from simple, probablilistic, and hierarchical cell-graphs, as well as a hybrid combination of simple and hierarchical approaches. Similarly, Ali et al [21], utilized attributes of probabilistic cell-cluster graphs for differentiating oropharyngeal cancers. These subgraphs offer the advantage of being able to explicitly and independently model spatial architecture of nuclei and glands within the epithelial and stromal regions.

In this paper, we describe a new QH methodology which aims to utilize the directionality of glands and associate disorder in gland orientations to predict the degree of malignancy and subsequently the risk of post-surgical biochemical recurrence in CaP patients. The hypothesis is that normal benign glands align themselves with respect to the fibromusclar stroma, and thus display a coherent directionality. Malignant prostate glands, however, lose their capability to orient themselves and display no preferred directionality. Additionally with increasing degree of malignancy and disease aggressiveness, the coherence of gland orientations within localized regions is completely disrupted. In other words, the entropy (which captures disorder) in gland orientations tends to increase as a function of malignancy.

The CGA features aim to capture the directional information in localized gland networks in excised histopathology sections to characterize differences in gland orientation between (a) malignant and benign regions and (b) CaP patients who do and do not experience biochemical recurrence following RP. The CGA methodology comprises the following main steps.

**Figure 1:**
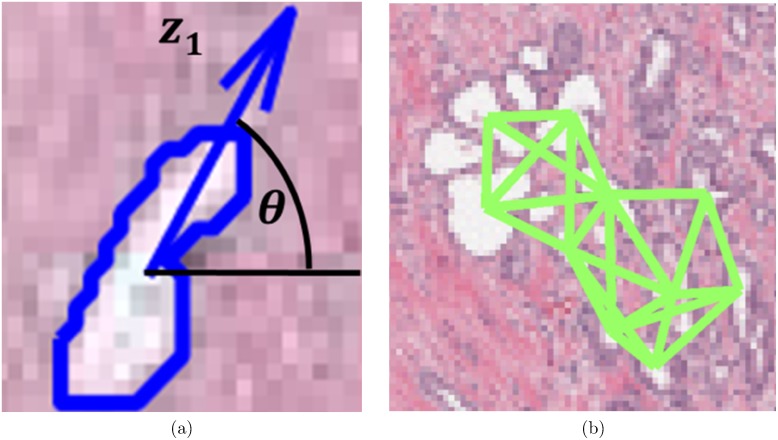
Gland characteristics of interest for calculating CGA features. (a) For each gland, the angle between the major axis of the gland (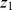) and the x-axis is calculated. (b) Subgraphs connect the centroids of neighboring glands into locally connected gland networks.

For CGAs, a segmentation algorithm is first employed to individually segment gland boundaries from digitized pathology sections. To each gland, we ascribe an angle that reflects the dominant orientation of the gland based off the major axis as shown in Figure 1(a). A subgraph is then constructed to link together glands proximal to each other into a gland network as illustrated in Figure 1(b). The subgraphs, unlike the graphs for Voronoi, Delaunay and minimum spanning trees that have been previously used to characterize global glandular architecture [26], allows for characterization of local gland arrangement. Use of local subgraphs prevent graph edges from traversing heterogeneous tissue regions such as stroma and epithelium. The co-occurrence matrix, previously used to characterize image intensity textures, is used to capture second-order statistics of gland orientations within each gland network in the image. Hence each co-occurrence matrix captures the frequency with which orientations of two glands proximal to each other co-occur. Co-occurrence features such as entropy are extracted from the co-occurrence matrix associated with each gland network to capture the degree to which proximal gland orientations are similar or divergent to each other. Hence a neighborhood with a high entropy value would reflect a high degree of disorder among gland orientations while a low entropy value reflects that the gland angles appear to be aligned roughly in the same direction.

Given that we expect to see glandular angle disorder in (a) malignant versus benign regions and (b) biochemical recurrence cases versus non-recurrence cases, second-order statistical angular features like entropy represent a novel, reproducible, and interpretable way to characterize disease appearance on histopathology. Unlike first order statistics of angles, the co-occurring gland angular features are able to implicitly capture the cyclical properties of gland orientation. The use of local subgraphs generated by a probabilistic decaying function help define local gland networks within which the CGA features can be extracted and analyzed.

In this work, we demonstrate the utility of CGA features for the following classification tasks: 1) differentiating cancerous and non-cancerous prostate tissue regions, and 2) distinguishing CaP patients with and without biochemical recurrence following radical prostatectomy.

**Figure 2:**
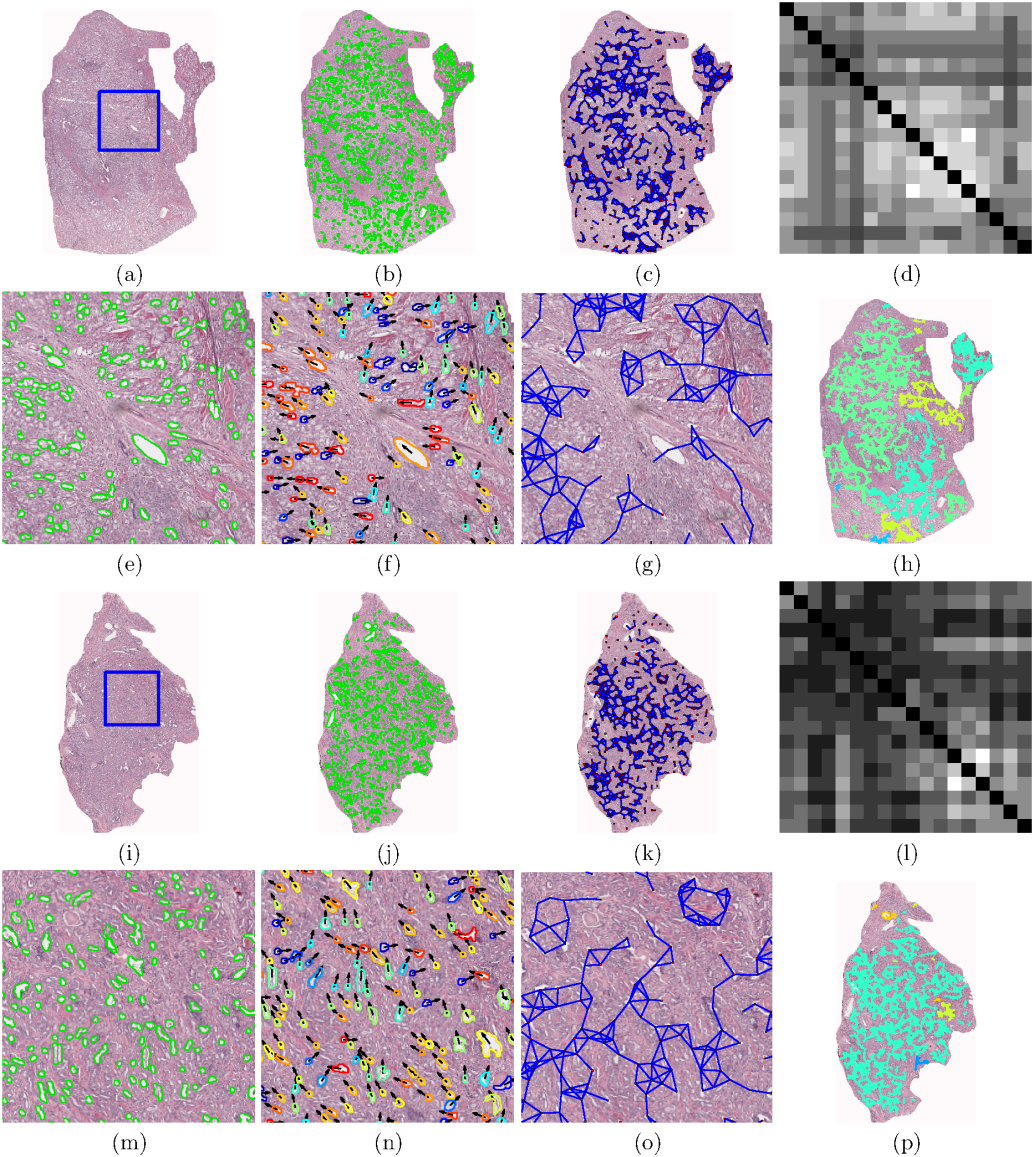
Annotated histological CaP regions ((a) and (i)) pertaining to a BCR (a)–(h) and a NR (i)–(p) case study, respectively. (b), (j) Automated gland segmentation of gland boundaries. (c), (k) Subgraphs showing connections between neighboring glands. An enlarged view of the boxed region in (a) and (i) respectively, illustrates (e), (m) segmented glands, (f), (n) gland angles, and (g), (o) gland network subgraphs. (f), (n) Arrows denote the directionality of each gland. Boundary colors (blue to red) correspond to angles 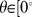 180°]. (g), (o) Localized gland networks define the region of each angular co-occurrence matrix. (d), (l) Summed angular co-occurrence matrices denote the frequency with which two glands of two directionalities co-occur across all neighborhoods (white elements reflect greater co-occurrence). Diagonal co-occurrence values have been omitted to provide better contrast compared to the off-diagonal components. (h), (p) Colormap of the gland subgraphs correspond to the intensity average in each neighborhood.

Figure 2 shows two representative studies: a biochemical recurrence (BCR) and a non-biochemical recurrence (NR) case. For the BCR case, we can see greater disorder in the gland orientation illustrated via the vector plot in Figure 2(f). The angle-based colormap for BCR characterizes the disorder in BCR cases, as evidenced by the a large spectrum of colors, each color representing a different orientation. Conversely, for the NR case, (Figure 2(n)), the colormap shows a smaller range of colors, suggesting less variance in the gland directionality. The gland directionality differences are also reflected in Figures 2(d), (l) via the angular co-occurrence matrix. The brightness of the off-diagonal elements of the matrix reflect greater co-occurrences of differentially oriented gland angles for the BCR case (Figure 2(d)) compared to the NR case (Figure 2(l)). These differences in the angular co-occurrence matrices are detected by the second order statistics, as Figures 2(h), (p) illustrate different color patterns based on the value of the statistics in each subgraph.

It can also be observed in Figures 2(c), (k) that subgraphs capture local gland neighborhoods. Since the subgraphs are localized and limited to the epithelial regions alone, the contributions from within the stromal regions are minimized. The CGAs therefore provide a compact, interpretable and quantitative representation of gland architecture and prostate cancer morphology which can be employed to distinguish (a) cancer from benign regions and (b) BCR from NR cases.

The remainder of this paper is structured as follows. We first introduce the theory and methodology for **CGA**s. **Materials and Methods** outlines the process of obtaining the study cohorts and provides details for workflow and comparative methodologies used in this study. **Experimental Results and Discussion** provides specific instances in which we test our CGA methodology. Lastly, **Concluding Remarks** discusses our overall contributions and future work.

## Quantitative Histomorphometry via Co-occurring Gland Angularity (CGA)

### Notation

We define an image scene as 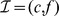, where the image scene 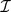 is described by a spatial grid 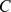 of locations 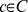, each of which are associated with a unique intensity value 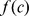. For intensity images 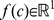 and for color images 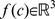. We define a sub-region, 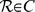, within the scene, where a subgraph 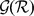 can be defined.

Each 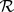 is comprised of a number of glands 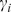, which are represented as nodes, 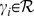, where 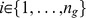, where 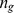 is the number of glands in 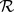. We can also define 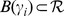 as the set of boundary points associated with gland 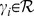.

Hence we can formally define 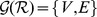 where 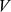 represents the set of glands 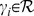 and 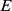 is the set of edges that connect 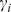 to other adjacent glands within 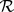. Each 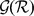 can then be represented via an attribute vector of CGA features 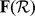. A classifier 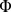 can then be trained to identify any 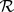 as belonging to one of two classes 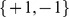. In this work, the classifier 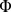 will be trained to distinguish each 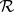 as (a) malignant or benign or (b) BCR or not.

### Calculating Gland Angles

To determine the directionality for each gland 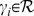, 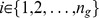, we perform principal component analysis [27] on a set of boundary points 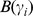 to obtain the principal components 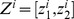. The first principal component 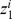 describes the directionality of gland 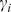 in the form of the major axis, along which the greatest variance occurs within 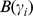. The principal axis 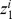 is expressed as vector, of a single directionality, and is defined as
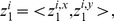
(1)where 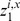 represents the direction in the 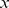-direction, and 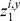 represents the direction in the 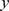-direction. 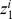 is subsequently converted to an angle 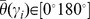 calculated counterclockwise from the vector 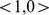 by 
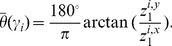
(2)A depiction of the process for estimating gland orientations is shown in Figure 1(a).

### Defining Local Subgraphs on Glands

Pairwise spatial relationships between glands are defined via sparsified graphs. For the subgraph 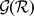 defined on region 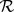, the individual edges can be defined between all pairs of 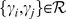 via a probabilistic decaying function described in Gunduz et al. [20], [23].
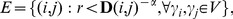
(3)where 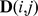 represents the Euclidean distance between 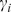 and 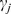. 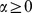 controls the density of the graph, where 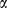 approaching 0 represents a high probability of connecting nodes while 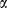 approaching 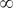 represents a low probability. 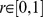 is an empirically determined edge threshold. An example of a resulting glandular subgraph network is shown in Figure 1(b).

### Gland Angularity Co-occurrence Matrices

The objects of interest for calculating CGA features are given by a discretization of the angles 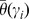, such that 
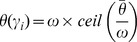
, where 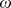 is an empirically derived discretization factor. Larger 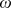 provide less specificity for counting co-occurring gland angles and smaller 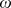 may not express co-occurring angles within the individual neighborhoods. The optimal 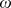 was chosen based off a 3-fold randomized cross-validation of parameters 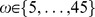. 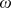 was set to be 10 in this work, allowing for angles to be discretized at every 10 degrees.

Neighbors defined by the local subgraphs 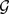, allow us to define neighborhoods for each 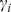. For each 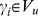, we define a neighborhood 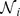, to include all 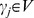 where a path between 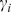 and 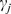, 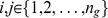 exists via 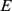 in the graph 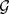.

An 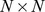 angular co-occurrence matrix 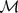 subsequently captures gland angle pairs, 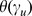 and 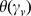, where 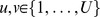 and 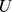 is the number of glands in 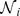, which co-occur within each neighborhood 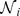. This can therefore be expressed in the following way.
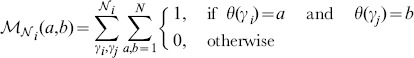
(4)where 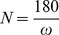, the number of discrete angular bins. An example of a angular co-occurrence matrix is shown in Figures 2(d) and (l).

### Second Order Gland Angle Statistics

We subsequently extract second order statistical features 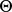 (Contrast energy, Contrast inverse moment, Contrast average, Contrast variance, Contrast entropy, Intensity average, Intensity variance, Intensity entropy, Entropy, Energy, Correlation, and two measures of information) from each angular co-occurrence matrix 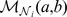. Formulations of these second order features 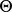 are described in Table 1. Figure 2 illustrates the visualization of the mean intensity measure of each 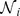 on a digitized histopathology image.

**Table 1:** Description of 13 CGA features.

CGA Feature ( 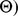		Description	Additional Notation
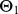	Contrast energy	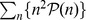	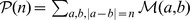
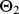	Contrast inverse moment	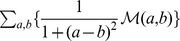	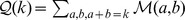
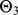	Contrast average	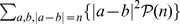	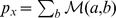
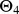	Contrast variance	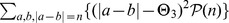	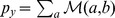
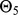	Contrast entropy	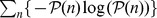	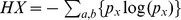
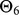	Intensity average	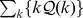	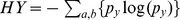
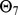	Intensity variance	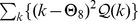	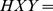
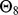	Intensity entropy	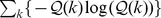	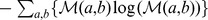
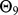	Entropy	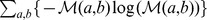	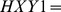
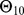	Energy	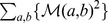	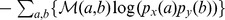
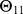	Correlation	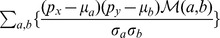	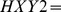
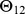	Information measure 1	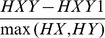	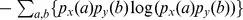
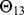	Information measure 2	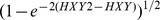	

## Materials and Methods

### Ethics Statement

Patients included in the study were obtained from independent sources. Cohort A was collected by Drs. Tomaszewski and Feldman obtained from IRB study “Analysis of Genetic Changes in Genitourinary Cancers” Protocol #707863. Cohort B was part the Score prostate project, run by Dr. Rebbeck, and approved by IRB study UPCC 13808 “Molecular epidemiology of prostate cancer” Protocol #36142. All IRB approval was obtained from the University of Pennsylvania, where the patient data was collected. Written consent was obtained for all patients for long term follow up. De-identified digital pathology samples and biochemical recurrence data used for this study was derived from data collected under informed consent. Since de-identified data was used, IRB consent was not required.

### Data Acquisition and Data Description

The datasets (obtained from the Hospital at the University of Pennsylvania) were comprised of 40 CaP patients who had undergone RP treatment. These studies were selected from a much larger cohort of over 3000 cases archived at the Hospital at the University of Pennsylvania. The cases were chosen to have an equal split of cases with BCR and NR following RP. Additionally, the search was limited to just Gleason scores 6–8 and pathologic stage pT2 and pT3.

**Figure 3:**
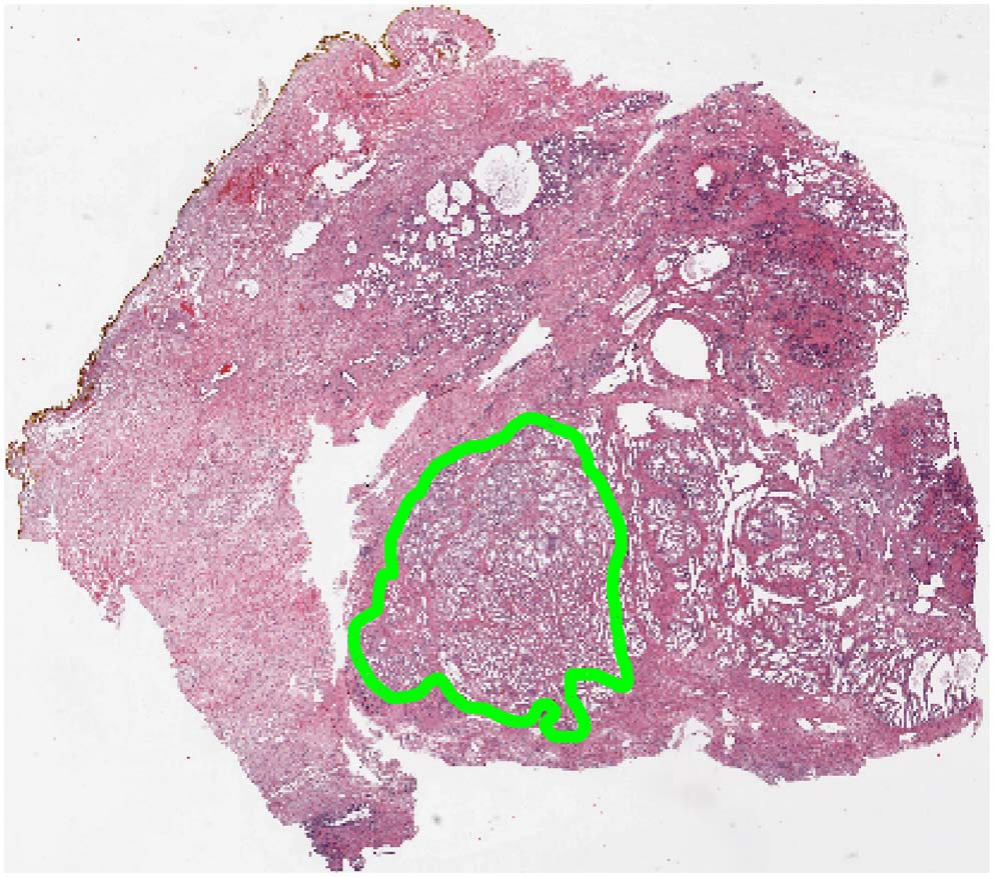
Workflow for building a CGA-based classifier. (a) Gland segmentation is performed on a region of interest. CGA methodology (highlighted within the dashed lines) leverages the gland segmentation to compute CGA features. (b) Angle calculation and (c) Subgraph computation is performed on the segmented image. (d) Angular co-occurrence matrix aggregates co-occurring gland angles within localized gland networks. (e) Mean, standard deviation and range of second order statistics (shown via differentially colored gland networks) create a set of CGA features for the region. (f) A CGA-based classifier can then be built using the features obtained from (e) to distinguish the categories of interest (either cancer versus benign regions or BCR versus NR).

For all CaP patients, following RP, the excised prostate was sectioned, stained with hematoxylin and eosin (H
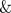
E), and digitized at a resolution of 0.5 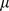
m per pixel or 20x magnification using an Aperio^®^ whole slide scanner. For each digitized image, CaP regions were annotated by a pathologist, as shown in Figure 3. 56 cancer regions were annotated across 40 patients, 28 from BCR patients and 28 from NR patients. Since no men without documented and biopsy confirmed prostate cancer undergo radical prostatectomy, there were no regions in this study which did not originate from a patient without cancer. Instead, 24 control regions were culled out from non-cancerous regions of the excised prostate for these patients.

### CGA Extraction Workflow

**Figure 4:**
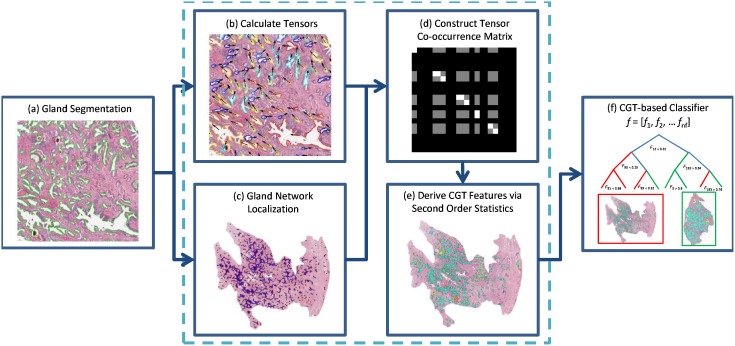
Annotation of a region of interest (shown in green) on prostate histopathology is performed by a pathologist. In this study, quantitative histomorphometric analysis is performed only in these regions.

As previously described in **Notation**, each region 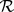 is characterized by a CGA features vector 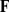. The 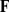 is used to train a machine learning classifier 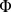 to distinguish between (a) cancerous from benign regions and (b) BCR from NR patients. The procedure for extracting 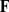 and training 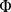 is described below and summarized by Figure 4.

#### Identification of glandular boundaries

**Figure 5:**
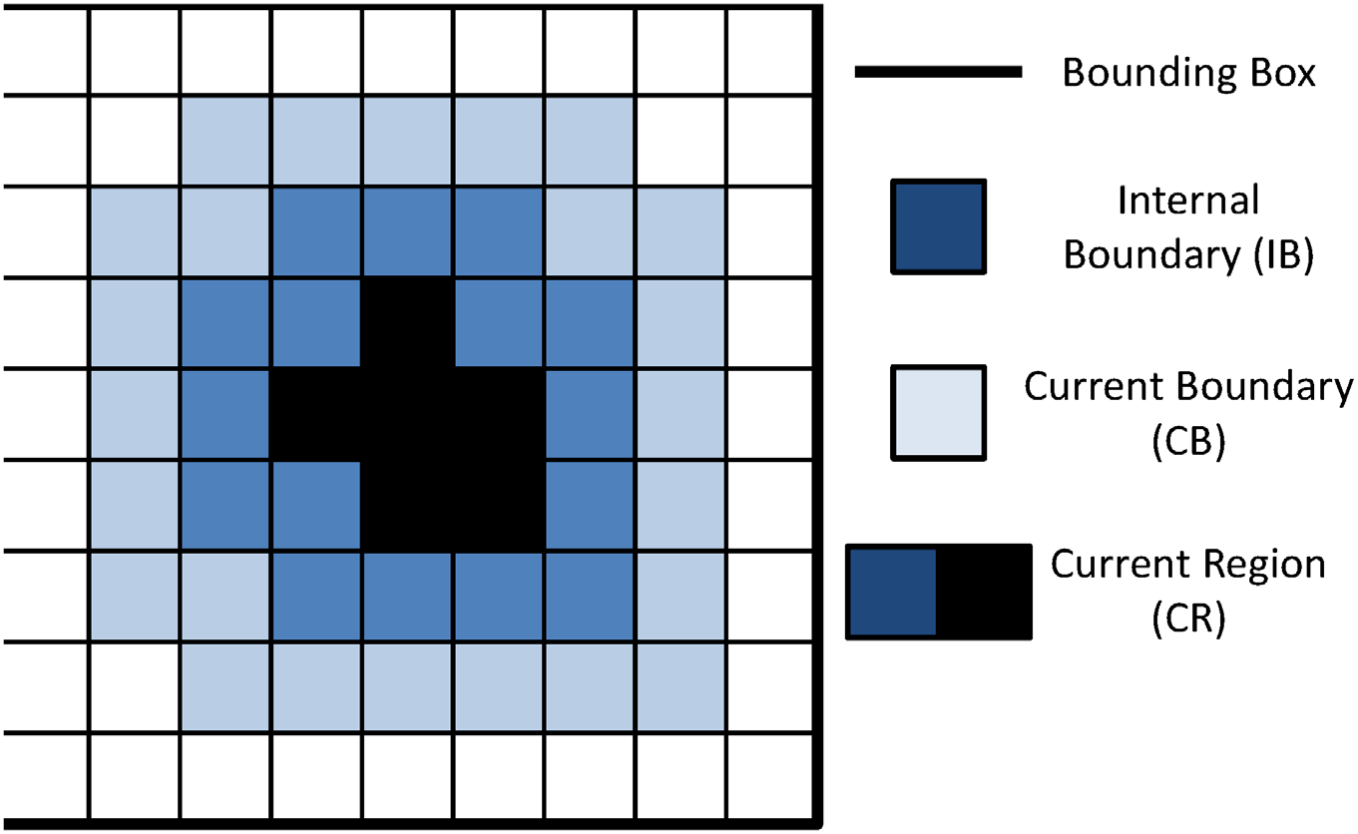
Schematic for region growing.

The detection and segmentation of gland boundaries is limited to only those regions manually annotated by the pathologist on the digitized histopathology sections. An automatic region-growing based prostate gland segmentation algorithm [13] is used to detect and segment glandular boundaries on the histological image as illustrated in Figure 5. Monaco et al. [13] previously was able to accurately identify prostate cancer regions at 93% accuracy via the gland segmentation procedure described below. Segmentation is performed using the luminance channel in CIELAB color space. In the luminance channel, glands appear as contiguous, high intensity pixel regions bordered by sharp edges as boundaries. To identify glands, the luminance image is convolved with a Gaussian kernel at multiple scales 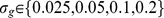 mm to account for multiple gland sizes. The peaks (maxima) of the resulting smoothed luminance images are used as seeds for a region growing procedure briefly outlined below.

1. A 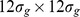 bounding box is initialized around each initial seed pixel, which represents the current region (CR), with 8-connected pixels surrounding CR, denoted as the current boundary (CB).
2. Next, the pixel in CB with the highest intensity is removed from CB and incorporated into CR. The 8 surrounding pixels of this new CR pixel, which are not already in CR, are incorporated into CB.
3. The boundary strength is identified at each iteration as shown in Figure 5. We define the internal boundary (IB) as all CR pixels adjacent to CB. Boundary strength is defined as the mean intensity of the pixels in IB minus the mean intensity of the pixels in CB.
4. Steps 2 and 3 are repeated until the algorithm attempts to add a pixel outside the bounding box.
5. The optimal region is defined as region CR at the iteration where maximum boundary strength was achieved.

Overlapping regions are subsequently resolved by removing the region with the lowest boundary strength. An example of our results can be seen in Figure 4(b).

#### CGA feature extraction

Based on the gland segmentation detailed in the section, **Identification of Glandular Boundaries**, CGA features are calculated as described in the previous section, **Quantitative Histomorphometry via Co-occurring Gland Angularity (CGA)**. The optimal parameters were chosen based off a 3-fold randomized cross-validation procedure for 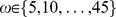 and 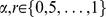. The best combination was found to be 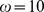, 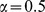 and 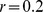, which was used to build the angle co-occurrence matrix for all cases in these experiments. Mean, standard deviation, and range of the CGA features described in the section **Second Order Gland Angle Statistics** are calculated. This yields 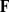 which is a set of 39 CGA features.

#### Building a CGA-based classifier

For each classification task (see **Experimental Results and Discussion**), a training set of positive and negative categories were compiled from the set of 40 cases (see Table 2). The training set was used to train a classifier 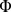 in conjunction with 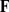 to distinguish between the categories of interest. The trained classifier 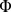 was then used to assign each region 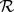 or study 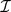 into classes 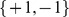 based on the classification tasks (a) distinguishing cancer from benign or (b) BCR from NR patients. 3-fold randomized cross-validation was used to train and evaluate classifier robustness. This involved randomly splitting the entire dataset into 3 equally sized sets with 2 subsets used for 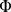 training and 1 subset used for independent evaluation. This procedure was repeated 100 times. In all our experiments a random forest classifier (a boostrapped aggregation of multiple decision tree classifiers) was used. We refer the reader to [28] for additional details on the random forest classifier.

**Table 2:** Overview of clinical datasets employed in this study.

	Clinical Variables	Cohort A (20)	Cohort B (20)	Combined Cohort (40)
**Pathological Gleason Score**	3+3	4 (20%)	1 (5%)	5 (12.5%)
	3+4	7 (35%)	17 (85%)	24 (60%)
	4+3	7 (35%)	2 (10%)	9 (22.5%)
	3+5	1 (5%)	- (-)	1 (2.5%)
	4+4	1 (5%)	- (-)	1 (2.5%)
**Pathologic Stage**	pT2	8 (40%)	12 (60%)	20 (50%)
	pT3a	9 (45%)	6 (30%)	15 (37.5%)
	pT3b	3 (15%)	2 (10%)	5 (12.5%)

### Comparative Methodologies

In order to compare the performance of the CGA features for the different classification tasks described in **Experimental Results and Discussion**, we explicitly modeled and evaluated a nunber of other state of the art (a) QH features and (b) nomograms, described below.

#### Quantitative histomorphometric attributes

*Gland Morphology (* 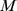 *):* Morphological descriptors [16] are extracted from the segmented glandular boundaries obtained in textbfIdentification of Glandular Boundaries Statistics such as the area ratio, perimeter ratio, and distance ratio are derived from the gland boundary information and the mean, standard deviation, median, and the ratio between the minimum and maximum values are calculated across all glands [16]. These features are summarized in Table 3.

**Table 3:** Summary of Quantitative Histomorphometric (QH) features to compare against CGA features as well as the number of features used to characterize each feature type.

Feature Type (QH)	Description	#
Gland Morphology	Area Ratio, Distance Ratio, Standard Deviation of Distance,Variance of Distance, Distance Ratio, Perimeter Ratio,Smoothness, Invariant Moment 1–7, Fractal Dimension,Fourier Descriptor 1–10 (Mean, Std. Dev, Median, Min/Max of each)	100
Voronoi Diagram	Polygon area, perimeter, chord length: mean,std. dev., min/max ratio, disorder	12
Delaunay Triangulation	Triangle side length, area: mean, std. dev.,min/max ratio, disorder	8
Minimum Spanning Tree	Edge length: mean, std. dev., min/max ratio, disorder	4
Glandular Density	Density of glands, distance to nearest gland	24
Co-occurrence Texture	Contrast energy, Contrast inverse moment, Contrast average,Contrast variance, Contrast entropy, Intensity average,Intensity variance, Intensity entropy, Entropy, Energy,Correlation, two measures of information: mean, std. dev.	26

**Figure 6:**
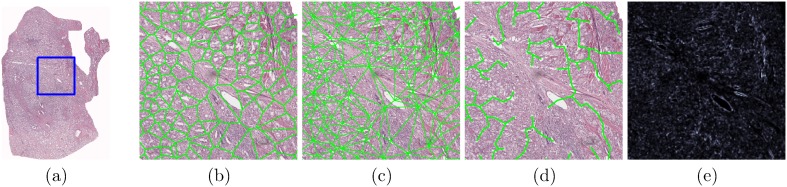
Examples of quantitative histomorphometric features for comparing against CGA features. QH features are extracted from (a) an annotated region on a digitized prostate histology slide following radical prostatectomy. Graphs for (b) Voronoi, (c) Delaunay, and (d) Minimum Spanning Trees as well as (e) a texture image feature are shown from the area denoted by a blue box in (a).

*Voronoi Diagram (* 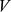 *):* Voronoi diagrams divide each region 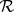 into non-overlapping polygons, each associated with a gland 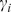, where each edge bisects two neighboring gland centroids 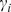 and 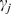, where 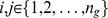. An example of the Voronoi Diagram constructed on top of a digitized pathology image with gland centers serving as nodes/vertices is shown in Figure 6(b). Statistics (See Table 3) such as the area, perimeter and chord length are recorded for each polygon and the average, standard deviation, disorder, and the ratio between the minimum and maximum values are calculated across all polygons in the image.

*Delaunay Triangulation (* 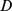 *):* Delaunay triangulation divides the image 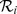 into triangles whose edges connect the gland centroids 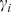 and 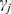 with 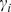 and 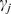 serving as the graph vertices. An example of the Delaunay triangulation graph is shown in Figure 6(c). Delaunay triangulation is related to the Voronoi diagram in that for each polygon in the Voronoi diagram, there is an accompanying edge which connects its gland centroid 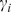 with 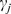 of an adjacent polygon. Edge length and area are computed for each triangle and the mean, standard deviation, minimum to maximum ratio, and disorder statistics are calculated across all triangles (see Table 3).

*Minimum Spanning Tree (* 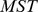 *):* MSTs are another graph representation where all gland centroids 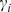 are connected with a minimum total edge weight defined as 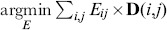, where 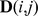 is the Euclidean distance between 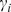 and 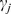. An example is shown in Figure 6(d). The average, standard deviation, disorder, and minimum to maximum ratio statistics are calculated across all edges in the graph (see Table 3).

*Gland Density (* 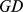 *):* GD features encompass two types of features. The first set of features denotes the number of 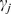 which lie within a 10, 20, 30, 40, and 50 pixel radius of each 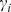. The second set of features denoted the distance between each 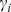 and its 3, 5, and 7 nearest centroids 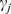. The average, standard deviation, and disorder for each of these features are computed across all glands (see Table 3).

*Co-occurrence Textures (* 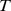 *):* Second order co-occurrence features are calculated from a symmetric co-occurrence matrix which aggregates the frequency with which two pixel intensities co-occur within a pre-determined spatial distance around each pixel 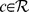. The size of the co-occurrence matrix is determined by the maximum possible intensity value in the image, which for 8-bit images is 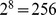. A spatial distance of 1 pixel was chosen for our experiments. For each 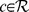, the second order co-occurrence features described in [15] are computed from the co-occurrence matrix. The mean and standard deviation across all 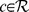 are used to build a single texture feature descriptor for each 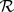.

#### Risk assessment nomogram and scoring systems

*Kattan Nomogram (* 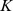 *):* The Kattan nomogram [8] was one of the earliest prediction tools to be developed for predicting biochemical failure following radical prostatectomy. Clinical predictors for the Kattan nomogram include 1) Pre-operative PSA, 2) Gleason Sum, 3) Primary Gleason score, 4) Surgical Margins, 5) Prostate Capsular Invasion, 6) Seminal Vesicle Invasion (SVI), and 7) Lymph Node Involvement. A raw score 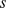, 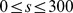 (higher score reflects higher risk of BCR) is derived from these predictors and risk for each patient is assessed in terms of a probability of being BCR free for a particular time interval following surgery.

*Stephenson Nomogram (* 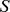 *):* The Stephenson nomogram [9] was developed along with Michael Kattan to incorporate the year of surgical intervention for predicting BCR. Based on 1) year of Radical Prostatectomy, 2) surgical margins, 3) extraprostatic extension (EPE), 4) seminal vesicle invasion (SVI), 5) lymph node involvement, 6) primary gleason grade, 7) secondary gleason grade, and 8) pre-operative PSA. A raw score 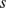, 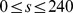, (higher score pertains to higher risk of BCR) is derived from these clinical features. Risk for each patient is assessed in terms of a score related to the probability of being BCR free for a particular time interval following surgery.

*Cancer of the Prostate Risk Assessment (* 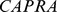 *):* The University of California in San Francisco (UCSF) CAPRA test [10], is based on overall score 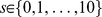, where 10 represents the highest risk of BCR. Clinical predictors for CAPRA include 1) Age, 2) Pre-operative PSA, 3) Primary Gleason, 4) Secondary Gleason scores, 5) Tumor Stage, and 6) Percent Positive Biopsy Cores.

*Memorial Sloan-Kettering Cancer Center (MS-KCC) Nomogram (* 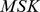 *):* One of the most popular nomograms with contributions from Kattan and Stephenson is the MS-KCC nomogram [8], [9]. The MS-KCC nomogram incorporates 1) Pre-Treatment PSA, 2) Age, 3) Primary Gleason Grade, 4) Secondary Gleason Grade, 5) Gleason Sum, 6) Year of Prostatectomy, 7) Months Free of Cancer, 8) Surgical Margins, 9) Extra Capsular Extension, 10) Seminal Vesicle Involvement, and 11) Lymph Node Involvement. Risk for each patient is assessed in terms of a score related to the probability of being BCR free for a particular time interval following surgery.

#### Classification accuracy

The predictive value of each classifier, obtained with the CGA features and comparative methodologies, is evaluated via classification accuracy 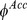 and via the area under the receiver operating characteristic curve (AUC) 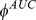. The Wilcoxon Rank Sum Test [29] was utilized to determine statistical significance between the classification performance of the QH features listed in Table 3 and the CGA features.

Classification Accuracy (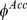) measures the ability of a classifier to correctly predict class label for 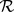 or 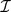 within the independent testing set and is calculated as
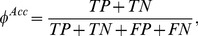
(5)where *TP*, *TN*, *FP*, and *FN* refer to the true positive, true negative, false positive, and false negative detection rates associated with 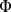 respectively.

#### Receiver operating characteristic


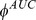
 represents an overall measure of predictive value for classifier 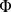, independent of the decision threshold. The receiver operative characteristic (ROC) curve is constructed by computing 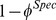 and 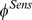 at each decision threshold where 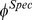 and 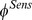 are defined as
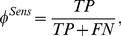
(6)


and
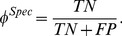
(7)


The AUC represents the area under the ROC curve, where an AUC of 1 reflects perfect classification and an AUC of 0.5 suggests that the classifier is performing no better than random guessing.

#### Kaplan-meier analysis

Kaplan-Meier analysis [30] is used to compare the BCR-free survival time between positive and negative control groups. In this study, the two groups are determined by a predictor 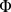. When plotted onto time versus BCR-free survival rate, the BCR free survival rate of the group will decrease at the time when a patient develops BCR. Thus, we expect the curve for the set of patients predicted to have BCR to drop quickly while the set of patients predicted as NR should remain BCR-free with the corresponding Kaplan-Meier curve not dropping off. The quantitative difference between the survival outcome can be determined via the logrank test [31]. The non-parametric test yields a 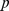-value, where lower 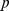-values denote greater significance between the survival distributions.

## Experimental Results and Discussion

We compare the performance of the CGA features versus the other QH (see Table 3) and risk assessment tools (see **Risk Assessment Nomogram and Scoring Systems**) in the context of the following experiments.

1. Distinguishing cancerous versus non-cancerous regions.
2. Predicting biochemical recurrence versus non-recurrence in CaP patients following RP.
3. Comparing BCR prediction of CaP patients following RP via CGAs against QH and risk assessment tools in terms of ROC and Kaplan-Meier analysis.

Each experiment and accompanying results are described in detail below and were conducted in accordance with the CGA extraction workflow described in the previous section.

### Experiment 1: Distinguishing Cancerous versus Non-cancerous Regions

80 regions were annotated by expert pathologists pertaining to 56 cancerous regions and 24 non-cancer regions. We compare the efficacy of CGA features with the QH features described in Table 3 for the purpose of differentiating cancerous regions from non-cancerous regions. For each set of CGA or QH features, a classifier was trained to distinguish between the cancerous and non-cancerous regions as previously described in **Building a CGA-based classifier**.

Mean and standard deviation for 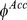 and 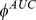 for the different QH and CGA features are shown in Table 4. CGA shows statistically significant (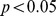) improvement in terms of 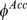 and 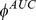 compared to all QH features. In fact the CGA features yield a near perfect classification performance for this particular task.

**Table 4:** Mean and Standard Deviation of (a) 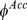 and (b) 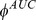 for QH features in distinguishing cancer from non-cancer regions over 100 runs of randomized 3-fold cross validation with a random forest classifier.

	GlandMorphology	Voronoi	Delaunay	MinimumSpanning Tree	GlandDensity	Texture	CGA
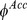	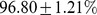	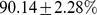	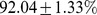	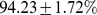	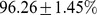	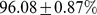	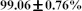
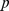 -value	3.2183e-28	2.1923e-35	1.0692e-35	7.0137e-35	8.3012e-31	4.6573e-35	-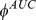	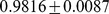	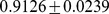	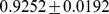	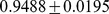	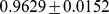	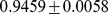	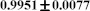
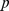 -value	7.1411e-23	2.2052e-34	2.2058e-34	3.9028e-34	7.4428e-31	2.1993e-34	-

Associated Wilcoxon Rank Sum Test 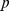-values for 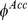 and 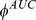 of the QH and CGA features.

### Experiment 2: Identifying CaP Patients with Biochemical Recurrence following Surgery

For each of 40 patients, the largest annotated cancer region for each of the 40 patients was selected. We compare the efficacy of CGA features with other state of the art QH features (Table 3) for differentiating patients who will develop BCR from those who will not, following RP. Similar to the procedure described in Experiment 1, the CGA and QH features were used to train classifiers to distinguish the BCR and NR cases over 100 runs of 3-fold cross-validation.

As illustrated in Table 5, CGAs outperformed each of the 6 other QH features in predicting BCR in 40 CaP patients in terms of 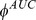 and 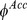. These results were statistically significant (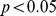). Not only do these results suggest that the CGA features were able to outperform other QH features, they also seem to suggest that in more aggressive CaP, disorder in glandular orientations appears to progressively increase.

**Table 5:** Mean and Standard Deviation of (a) 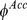 and (b) 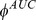 for QH features in distinguishing BCR from non-recurrence patients over 100 runs of randomized 3-fold cross validation with a random forest classifier.

	GlandMorphology	Voronoi	Delaunay	MinimumSpanning Tree	GlandDensity	Texture	CGA
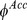	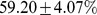	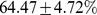	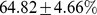	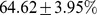	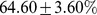	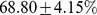	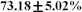
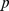 -value	5.418e-10	1.2662e-08	1.1547e-10	1.7115e-11	8.4514e-10	6.9014e-28	-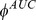	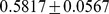	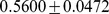	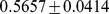	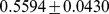	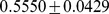	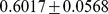	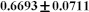
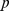 -value	8.4424e-08	3.6478e-06	1.453e-07	4.0656e-09	4.9759e-11	0.012387	-

Associated Wilcoxon Rank Sum Test 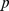-values for 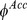 and 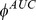 of the QH and CGA features.

### Experiment 3: Comparing CGA Features versus Risk Assessment Tools

The data was collected from two independent sources: Cohort A from the department of Pathology and Laboratory Medicine at the University of Pennsylvania and Cohort B from the department of Clinical Epidemiology and Biostatistics at the University of Pennsylvania. Of the 40 patients, only 20 patients in Cohort B had associated clinical variables for nomogram prediction of BCR. A further breakdown of the data is summarized in Table 2. We utilize this independent data collection to perform an independent study with a larger predicted training set compared to the 3-fold cross-validation performed in the other experiments.

Similar to the CGA classifier, a nomogram based classifier is also constructed based off the corresponding risk scores, 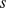. For the results in Table 6, training and evaluation of these classifiers was performed as described in Experiments 1 and 2 within Cohort B.

**Table 6:** Mean and Standard Deviation of (a) 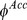 and (b) 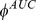 for predicting BCR over 100 runs of randomized 3-fold cross validation via Random Forest classifiers.

CaP Predictor	Kattan	Stephenson	CAPRA	MS-KCC	CGA
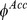	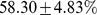	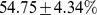	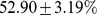	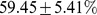	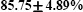
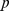 -value	4.2558e-35	3.3989e-35	1.3793e-35	5.2764e-35	-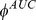	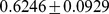	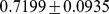	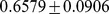	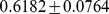	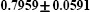
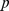 -value	5.1173e-25	6.0125e-10	2.1506e-21	7.2366e-29	-

Associated Wilcoxon Rank Sum Test 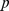-values for 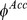 and 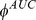 of each risk assessment tool compared to CGA features for predicting BCR are shown.

CGAs outperformed 4 state-of-the-art prostate cancer nomograms, demonstrating 85% classification accuracy compared to 59% accuracy for the MS-KCC nomogram, which had the second highest 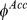 (Table 6). CGAs showed statistically significant improvement in 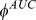 over all nomograms. Perhaps the most significant message in the results shown in Table 6 is that the CGA features in the absence of clinical variables PSA, Gleason score, stage, etc. were still able to outperform the 4 nomograms.

To increase the size of the testing set to 20 patients, we performed a study using two independent cohorts. Analysis via the calculation of the Receiver Operating Characteristic (ROC) curve is used to determine the overall performance across all classification thresholds of each classifier 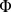.

For the results in Table 7, we perform the classification on Cohort B by creating a classifier 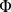 trained from Cohort A. For the Random Forest classifier, each prediction is given a fuzzy decision value 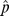 between −1 and 1. Nomograms do not require further training beyond the original fitting done in the original study from which the nomogram was developed. Each nomogram is designed to predict BCR risk based on a score 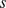. By setting decision thresholds at different 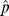 and 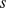 for 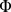 respectively, we obtained sensitivity and specificity scores at each threshold. The area under the ROC curve (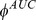) is subsequently calculated for each 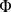 to compare their performance in predicting BCR within Cohort B.

**Table 7:** Independent validation of 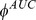 for predicting BCR from non-recurrence CaP patients.

CaP Predictor	Kattan	Stephenson	CAPRA	MS-KCC	CGA
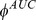	0.5500	0.5600	0.6050	0.5850	**0.7600**

CGA features demonstrate a clear improvement over 4 state-of-the-art nomograms, showing an AUC of 0.76 compared to AUCs near 0.5 for the comparative nomograms. The weak performance on this independent cohort suggests the difficulty that these nomograms may have in predicting the risk of BCR for men with intermediate-risk Gleason scores and suggests that the addition of QH features could improve upon the current nomogram standards.

This training and testing procedure was repeated for Kaplan-Meier analysis of 20 patients within Cohort B. Kaplan-Meier surves demonstrate the difference in BCR free survival time associated with each risk assessment tool. The logrank test was used to determine a 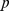-value associated with the difference between the survival curves. A lower 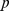-value indicates greater differences in the BCR-free survival between the predicted BCR and NR groups.

**Figure 7:**
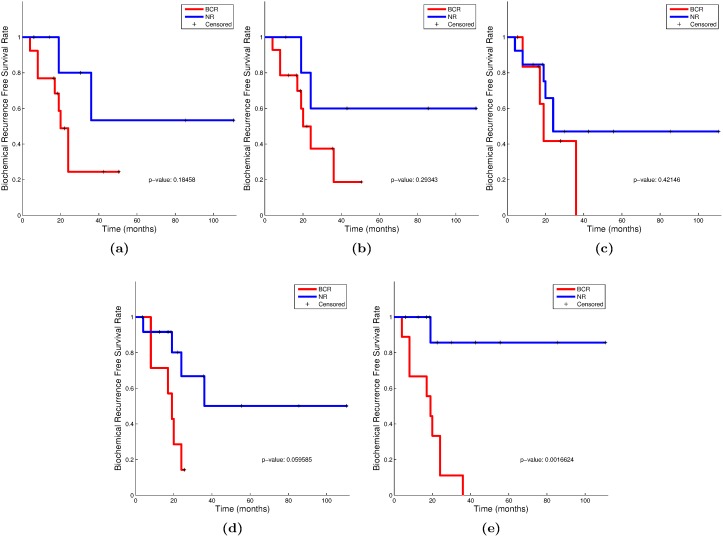
Comparison of Kaplan-Meier BCR-free survival curves differentiated via (a) Kattan nomogram, (b) Stephenson nomogram, (c) UCSF-CAPRA, (d) MS-KCC nomogram, and (e) CGA classifiers on an independent 20 patient cohort. Lower 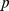-values are indicative of better predictors of BCR.

In Figure 7, we show Kaplan-Meier survival curves based on the predicted BCR and NR groups of each 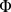. Based on the logrank test (shown in Table 8), patients were best differentiated via the CGA features, with a 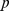-value of 0.0016 compared to 0.0596 for the MS-KCC nomogram, which had the next lowest 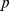-value. The CGA features represent the only feature set which show statistically significant differentiation (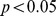) in the survival outcomes of its predicted patient cohorts.

**Table 8:** Logrank test 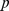-values for comparison of Kaplan-Meier survival curves of Cohort B stratified into BCR and NR groups by CGA features and CaP risk assessment tools.

CaP Predictor	Kattan	Stephenson	CAPRA	MS-KCC	CGA
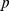 -value	0.1846	0.2934	0.4215	0.0596	**0.0016**

The most significant 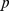-value is shown in **bold**.

## Concluding Remarks

In this paper, we present a novel set of quantitative histomorphometric features, co-occurring gland angles (CGAs), calculated on local subgraphs. CGAs represent a novel combination of subgraphs, gland angles, and angular co-occurrence matrices to quantify the local disorder in the gland angles on histopathology. For a cohort of 40 patients with Gleason scores 6–8 and treated with radical prostatectomy, we found CGA features demonstrated a statistically significant (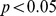) improvement in classification accuracy in distinguishing (a) cancer from benign regions and (b) biochemical recurrence from non-recurrence patients following surgery compared to 6 other state of the art QH features. Furthermore, we found CGAs to outperform 4 state-of-the-art postoperative nomograms for predicting BCR in CaP patients. Kaplan-Meier analysis of the independent cohort of prostate cancer patients with intermediate-risk pathological Gleason scores (via the logrank test) demonstrated that only the CGAs showed a statistically significant (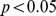) difference in predicting the survival distributions.

We do however acknowledge that our work did have its limitations. Firstly, even though randomized cross-validation strategies were employed to remove bias in the classifier, it would be optimal to validate our classifier on an independent testing set. Additionally, we also wish to control for the clinical variables used in this study (grade, stage, PSA levels, etc.) to further remove potential noise in the study cohorts. We hope to address these limitations in future work. An immediate next step will be to evaluate the performance of the CGA features in predicting aggressive disease from needle core biopsies alone as opposed to prostatectomy specimens.
